# Cross-Linking Strategies for Electrospun Gelatin Scaffolds

**DOI:** 10.3390/ma12152476

**Published:** 2019-08-04

**Authors:** Chiara Emma Campiglio, Nicola Contessi Negrini, Silvia Farè, Lorenza Draghi

**Affiliations:** 1 Department of Chemistry, Materials and Chemical Engineering “G. Natta”, Politecnico di Milano, via Mancinelli 7, 20131 Milan, Italy; 2 INSTM, National Interuniversity Consortium of Materials Science and Technology, Local Unit Politecnico di Milano, Piazza Leonardo da Vinci 32, 20133 Milan, Italy

**Keywords:** gelatin, cross-linking, electrospinning, scaffold, nanofibers, natural polymers, tissue engineering, regenerative medicine, soft tissues

## Abstract

Electrospinning is an exceptional technology to fabricate sub-micrometric fiber scaffolds for regenerative medicine applications and to mimic the morphology and the chemistry of the natural extracellular matrix (ECM). Although most synthetic and natural polymers can be electrospun, gelatin frequently represents a material of choice due to the presence of cell-interactive motifs, its wide availability, low cost, easy processability, and biodegradability. However, cross-linking is required to stabilize the structure of the electrospun matrices and avoid gelatin dissolution at body temperature. Different physical and chemical cross-linking protocols have been described to improve electrospun gelatin stability and to preserve the morphological fibrous arrangement of the electrospun gelatin scaffolds. Here, we review the main current strategies. For each method, the cross-linking mechanism and its efficiency, the influence of electrospinning parameters, and the resulting fiber morphology are considered. The main drawbacks as well as the open challenges are also discussed.

## 1. Introduction

Electrospinning (ES) is a well-established fabrication technique to produce sub-micron, non-woven fibers from polymer solutions (more rarely, from melts, sols, and emulsions) [1,2]. The working principle of ES relies on the application of an electric field to stretch and solidify a polymer solution and eventually collect it on a target to obtain the electrospun matrix/scaffold. In more detail, the polymer solution is generally introduced in the electric field through a charged metal capillary (i.e., spinneret) fed by a syringe pump; a grounded or oppositely charged target (i.e., collector) is placed to a set distance. When the force of the electric field overcomes the surface tension of the polymer solution, the so-called “Taylor cone” develops at the tip of the spinneret and a fine jet is ejected. As the polymer jet travels from the spinneret to the collector, the solvent gradually evaporates. Bending instability causes the jet to whip and further stretch. If the process is correctly tuned, almost dried ultra-fine fibers are collected on the target as a non-woven scaffold.

ES is an excellent candidate process to fabricate tissue engineered scaffolds, as the resulting electrospun matrices possess many desired properties for cell and tissue growth. Electrospun non-wovens, in fact, can closely resemble the architecture of extracellular matrix (ECM). Compared to other fabrication techniques capable of processing biomolecules into fiber scaffolds (e.g., molecular self-assembly, thermally induced phase separation), ES has attracted significant interest for being a relatively simple but extremely versatile process [3]. By adjusting processing parameters (i.e., applied voltage, spinneret-collector distance, and solution flow rate) and solution parameters (i.e., polymer concentration, solution conductivity, and solvent), the fiber morphology and scaffold properties can be tuned [4]. Additionally, different collector geometries can be used (e.g., stationary flat, rotating cylindrical, or grid) to collect fibers with a specific orientation or to produce scaffolds with specific 3D geometries [5].

Most soluble polymers, both synthetic and natural-derived, can be processed and a large variety of materials have been successfully electrospun into fibers with diameters down to 20 nm [6,7]. Naturally occurring polymers are often chosen as they show high biocompatibility, a favorable pro-remodeling host immune response and an instructive micro-environment for tissue-remodeling [8]. However, despite the above-mentioned advantages, processing natural-derived polymers via electrospinning is significantly more challenging compared to synthetic materials. ECM derived polymers (e.g., collagen, elastin, fibrinogen and hyaluronic acid), in particular, show poor solubility and the viscoelastic properties of the polymer solution are frequently inadequate to guarantee a stable and continuous process [9]. Gelatin represents an excellent option to prepare electrospun scaffolds for tissue engineering, thanks to the possibility of maintaining cell-adhesive motifs and, at the same time, of easily fabricating nanofibrous structures. However, for its application at body temperature, an adequate cross-linking procedure must be chosen to stabilize the electrospun matrix while preserving its favorable properties. Here, we review recent works that describe the preparation by electrospinning of gelatin scaffolds for tissue engineering, focusing on their cross-linking strategies. We also report on the biomedical applications of electrospun cross-linked gelatin matrices, and critically discuss current achievements and open challenges in the field.

## 2. Gelatin

The use of gelatin is well established in a variety of applications including the food industry, the pharmaceutic industry, and cosmetics manufacturing. In the last decades, gelatin has become one of the most investigated natural-derived polymer in the biomaterials and tissue engineering fields [10].

**Figure 1:**
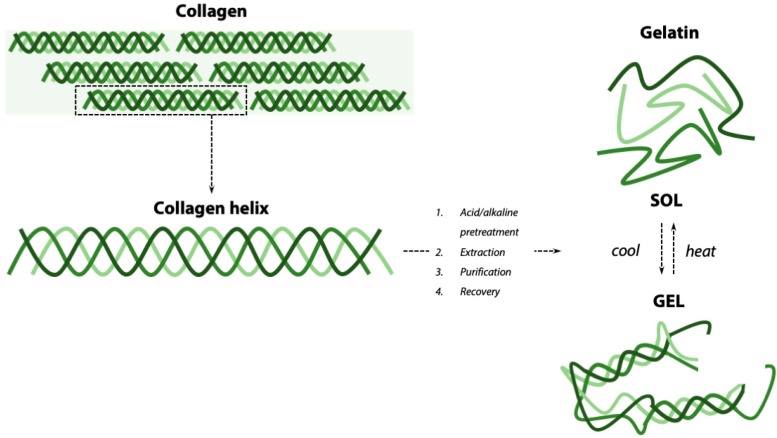
Processing of collagen for gelatin extraction. Collagen, characterized by a triple-helix structure and insolubility, is processed either by acid (gelatin A) or alkaline (gelatin B) pre-treatment. After extraction, purification, and recovery, gelatin, a soluble product, is obtained. When dissolved in water, gelatin undergoes a reversible sol-gel transition by heat–cool process.

Gelatin is a soluble protein derived from the partial hydrolysis of collagen (Figure 1), an insoluble fibrous protein and main constituent of the ECM in animal tissues, including skin, cartilage, and bone [11]. The collagen molecule is a right-handed bundle, composed by three parallel left-handed α-chains; each chain is composed by the repetition of the amino acidic sequence Gly-Xaa-Yaa, where Xaa is mostly proline and Yaa is mostly hydroxyproline [12]. Collagen molecules are organized in superstructures named microfibrils (40 nm diameter) and fibrils (100–200 nm diameter), eventually assembled into the collagen fibers that constitute the ECM matrix (50–500 nm diameter) [13].

To obtain gelatin, collagen can be derived from different sources. The animal (e.g., porcine, bovine, fish), its age, and the type of collagen (e.g., type I, type II, and others) will influence the properties of the extracted gelatin. The manufacturing process to extract gelatin from collagen also influences gelatin properties, including its molecular weight and isoelectric point [14]. Gelatin extraction from collagen requires a pre-treatment to cleave the cross-links that stabilize the collagen structure. The pre-treatments can be alternatively based on an alkaline, acid, or enzymatic process. The most common are the alkaline and acid treatments. Acid pre-treatment results in gelatin with isoelectric point 8–9 (“type A” gelatin), while alkaline pre-treatment results in gelatin with isoelectric point 4–5 (“type B” gelatin) [11]. Gelatin is extracted from pre-treated collagen by immersion in salt solutions (e.g., sodium chloride, Tris-HCl, phosphates, or citrates) or in acid solutions (e.g., organic acids such as acetic acid, citric acid, lactic acid, or inorganic acids such as hydrochloric acid). Finally, the recovery process consists of several steps including filtration, evaporation, drying, grinding, and sifting and returns gelatin powder. By regulating temperature, pH, and process time, the degree of collagen converted into gelatin during this process can be optimized [15]. Gelatin dissolves in hot water and spontaneously forms gels on cooling (Figure 1), with a sol-gel transition at T < 20–30 °C, depending on gelatin type and concentration [16]. This reversible gelation is associated with the transition of the gelatin polymeric chains from random coil to a partial restoration of the triple helices of collagen (Figure 1) [17].

Compared to native collagen, gelatin is more soluble in water and has lower antigenicity and immunogenicity in physiological conditions [11]. Gelatin is also highly biocompatible, versatile, completely resorbable in vivo, inexpensive, and widely available [18]. As a collagen derivative, gelatin still retains outstanding properties for the cells-biomaterial interactions, including exposure of ligands (i.e., peptide motifs such as arginine-glycine-aspartic acid, RGD sequence) that promote cell attachment by integrin-mediated interactions, and target sites for cellular metalloproteinases (MPP) that allow for the in vivo biodegradation of gelatin and ECM remodeling.

The many favorable properties of gelatin have made this natural-derived polymer one of the most investigated materials for tissue engineering applications in a variety of forms, including porous scaffolds [19], microspheres [20], 3D printed scaffolds [21], and electrospun matrices [18]. Due to its solubility at body temperature (i.e., T = 37 °C), however, gelatin cannot be used as such for in vivo tissue engineering applications and a cross-linking mechanism is required to obtain structures with suitable properties and appropriate stability.

## 3. Cross-Linking Methods for Gelatin

**Figure 2:**
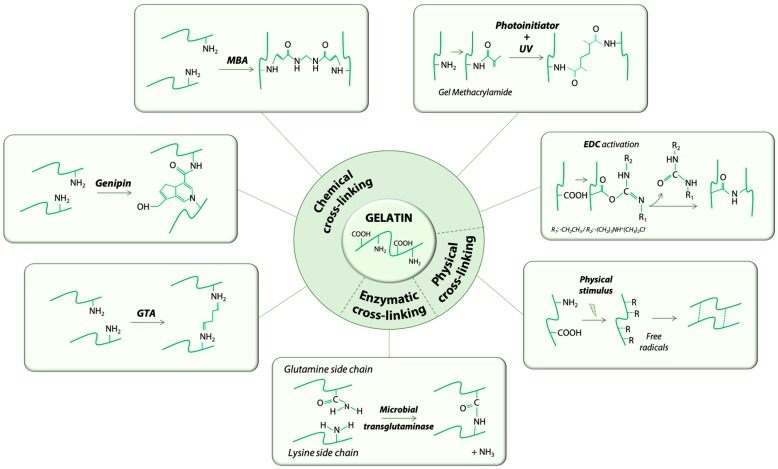
Schematic illustration of representative cross-linking methods used to fabricate gelatin hydrogels.

The cross-linking method chosen to stabilize gelatin structures for biomedical applications is crucial. Several scaffold properties, including degradation kinetic, mechanical and rheological properties, and biocompatibility can be substantially modified during this step. The cross-linking methods proposed so far can be divided into three main categories, physical, chemical, and enzymatic, as summarized in Table 1, and are described in detail in the following sections (Figure 2).

### 3.1. Physical Methods

Physical cross-linking methods mainly include the use of irradiation (i.e., high energy electron beam [22,23,24] or γ-irradiation [25,26]), plasma [27], and dehydrothermal treatment [28,29,30]. When a high energy electron beam is used to cross-link gelatin hydrogels (e.g., electron beam) the polymer chains scission might occur with consequent formation of free radicals that eventually form bonds between the gelatin polymer chains [22,53]. Plasma has been also adopted as a possible cross-linking method. In fact, when plasma is applied on gelatin solutions, free radicals (e.g., hydroxyls) can be formed after reaction of oxygen radicals with water to eventually form cross-linking bonds between gelatin polymer chains [27]. Differently, dehydrothermal (DHT) treatments are performed by prolonged (i.e., typically days) application of high temperatures under vacuum (T > 100 °C, *p* < 100 mTorr). The combined application of high temperature and low pressure promotes water condensation and removal from the gelatin polymers, thus promoting the formation of intermolecular cross-links to form hydrogels [54,55]. In general, physical cross-linking allows avoiding the addition of potentially toxic compounds in the polymer network as well as the use of solvents during the hydrogel preparation, which might eventually result in cytotoxic effects. Moreover, in some cases (e.g., γ-irradiation, electron beam), it is possible to achieve gelatin sterilization simultaneously with the cross-linking reaction. However, gelatin physical cross-linking methods are generally affected by a relatively lower cross-linking degree [56] and mechanically weaker hydrogels [57], compared to chemical cross-linking methods.

### 3.2. Chemical Methods

Chemical cross-linking involves the formation of covalent bonds between the gelatin polymeric chains, thus allowing the obtainment of more stable gelatin hydrogels with controllable physico-mechanical properties, compared to physical methods. Depending on the chemical cross-linking reaction, these methods can be divided in two main groups, (1) zero length and (2) non-zero length types.

In zero length methods, the cross-linker catalyzes the direct bonding between polymer chains but it is not included in the hydrogel network as it is removed at the completed reaction. The most widely used zero-length chemical cross-linking method for gelatin relies on N-ethyl-N′-(3-(dimethylamino) propyl) carbodiimide (EDC), with or without the addition of N-hydroxysuccinimide (NHS). EDC reacts with the carboxylic groups of aspartic and glutamic residues of gelatin molecule forming an intermediate (O-acylisourea) that undergoes nucleophilic attack by the amine lysine residues of gelatin to form amide bonds between the gelatin polymer chains. NHS can be added to the reaction to prevent the O-acylisourea intermediate hydrolysis. Amide bonds eventually formed by the EDC(/NHS) reaction form the gelatin hydrogel network [58]. The EDC/NHS cross-linking method has notable advantages, including a high conversion efficiency, mild reaction conditions, and excellent preservation of gelatin biocompatibility [59]. Many works describe the optimization and characterization of hydrogels produced by this method [31,34,60], with applications in the tissue engineering and regenerative medicine fields for the fabrication of peripheral nerve guides [61], drug release systems [32], and scaffolds for bone tissue healing [33].

Non-zero length methods involve the use of a cross-linker that is eventually incorporated into the polymeric network. A variety of cross-linkers have been proposed and investigated to obtain gelatin hydrogels with controllable and highly versatile properties. Most of the investigated cross-linkers react with the gelatin polymer chains to form covalent bonds between the gelatin amino groups. For example (Table 1), investigated cross-linkers include aldehydes (e.g., formaldehyde [35] and glutaraldehyde [36,37]), isocyanates [48], acrylamides [46,47], and epoxides [49]. Glutaraldehyde has been widely used as gelatin cross-linker. When added to a gelatin solution, the reaction between the gelatin amines and the carbonyl groups of glutaraldehyde leads to the formation of a gelatin hydrogel network incorporated with the glutaraldehyde cross-linker molecule [62]. Although glutaraldehyde has been shown to be an efficient cross-linker to obtain stable gelatin hydrogels [36,37], its use has raised some concerns in terms of safety and biocompatibility [63]. Accordingly, different alternative molecules were proposed; genipin and gelatin methacryloyl (with UV activation), for instance, are currently among the mostly investigated cross-linking methods in the biomedical field. Genipin [64] is a natural aglycone compound extracted from *Gardenia jasminoides Ellis*, used in traditional Chinese medicine. Despite its high cost, genipin has been intensely studied as cross-linker for protein-based hydrogels, thanks to the biocompatibility of the resulting product and the anti-inflammatory properties of genipin. The cross-linking reaction of genipin involves the ring opening of the genipin molecule by an amino group nucleophilic attack; then, a two-step reaction covalently binds genipin to the gelatin polymeric chains [65]. In the biomedical field, several works describe its use for gelatin-based biomaterials and scaffolds [38,39,40,41]. Alternatively, gelatin methacryloyl (commonly named GelMA) can be obtained by methacryloyl substitution of gelatin amino and hydroxyl groups. After gelatin modification by methacrylic anhydride, a photo initiator is added to the GelMA solution that is subsequently cross-linked by irradiation. The most commonly used photo initiator is Irgacure^®^ 2959 [42,43,44,45], combined with UV irradiation, since it allows for cell embedding in the gelatin solution during the cross-linking. Despite the cross-linking of GelMA is promoted by UV irradiation, we here classified GelMA as a non-zero length cross-linking method due to the insertion of methacrylic groups that are eventually incorporated into the gelatin hydrogel network, as for the non-zero length cross-linking method definition.

### 3.3. Enzymatic Methods

Transglutaminase, an enzyme found in many plant and animal species, can alternatively be used to promote the formation of covalent cross-linking bonds between the gelatin chains. Specifically, this enzyme catalyzes the acyl-transferase reaction between gelatin glutamine residues and gelatin primary amino groups. Typically, microbial transglutaminase (mTG), an innocuous enzyme commonly used in the food industry, is used to fabricate gelatin hydrogels (10–30 U/g of gelatin [66]) for biomedical applications. The use of mTG allows formation of gelatin hydrogels (i.e., typical required time 20–180 min [52]), stable in physiological-like conditions (i.e., aqueous environment at 37 °C) for weeks [52]. Alternatively, oxidoreductases such as tyrosinase have been proposed to successfully crosslink gelatin hydrogels [67], despite the fact that they are mainly reported in literature to crosslink gelatin/chitosan blends [68,69] due to the low tyrosine content in gelatin, which might limit the enzymatic crosslinking reaction efficacy. Enzymatically-cross-linked gelatin scaffolds have gained great interest, especially thanks to the possibility of fabricating cell-laden hydrogels, given the cytocompatibility of the enzymatic-driven cross-linking reaction that can be conducted in a cell-friendly environment [50,51,52].

## 4. Cross-Linking Strategies for Electrospun Gelatin Fibers

**Figure 3:**
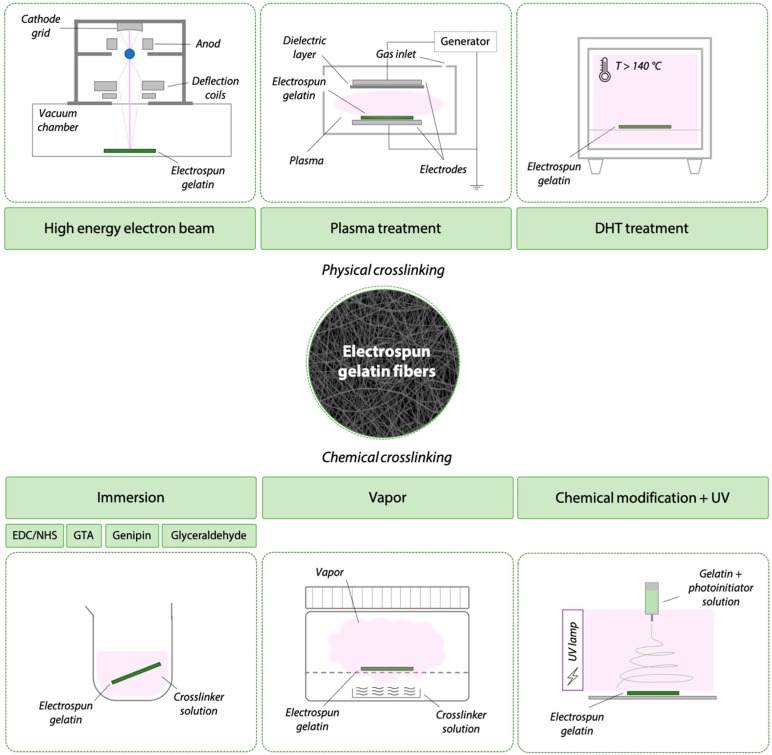
Schematic representation of cross-linking strategies used for the stabilization of electrospun gelatin matrices. Physical cross-linking can be performed by high energy electron beam, plasma treatment, or dehydrothermal treatment. Chemical cross-linking can be performed by immersion in a cross-linking solution, by using vapors of the cross-linker, or by chemically modifying gelatin to subsequently cross-link it by UV irradiation.

Electrospun matrices of gelatin in native form (i.e., not cross-linked) are water soluble and mechanically weak (i.e., in vivo condition T = 37 °C). During the necessary cross-linking of electrospun gelatin matrices for biomedical applications, promoting fiber stability and morphological maintenance is essential. The stabilization of ultrafine gelatin fibers by cross-linking requires a fine adjustment of conventional cross-linking methods (see Paragraph 3). The cross-linking of electrospun gelatin nanofibers can be achieved either by physical or chemical methods (Figure 3). These methods can either be applied upon the completed electrospinning process (i.e., post-processing cross-linking) or during the electrospinning process (i.e., in situ cross-linking). Physical cross-linking methods (Figure 3) involve the application of external stimuli (e.g., high energy electron beam, plasma, or dehydrothermal treatment) to electrospun matrices that are lodged in a specific apparatus. Chemical cross-linking methods (Figure 3) can be performed by the immersion of the electrospun gelatin matrices in cross-linking baths or vapor. The UV irradiation is here considered a chemical cross-linking method due to the chemical modification applied to gelatin (i.e., GelMA) prior to UV irradiation to allow the formation of covalent bonds among the polymer chains.

Examples of the above-mentioned cross-linking strategies, together with the main parameters involved in the stabilization of electrospun gelatin fibers and their biomedical applications, are summarized in Table 2. For each group (i.e., physical and chemical methods) and reported example, the parameters involved in the cross-linking strategy are highlighted together with the type of gelatin used for producing electrospun matrices and the final proposed application.

### 4.1. Physical Strategies to Cross-Link Electrospun Matrices

High energy electron beam, plasma treatment, and dehydrothermal treatment (DHT) are physical cross-linking strategies described for the stabilization of electrospun gelatin matrices (see Paragraph 3.1). Only few works describe these strategies, generally highlighting a lesser efficiency of physical methods compared to chemical ones [54,56,70,72]. As depicted in Figure 3, physical cross-linking mechanisms involve post-production processes as the cross-linking stimulus is applied after the gelatin matrix is fabricated by electrospinning.

**Figure 4:**
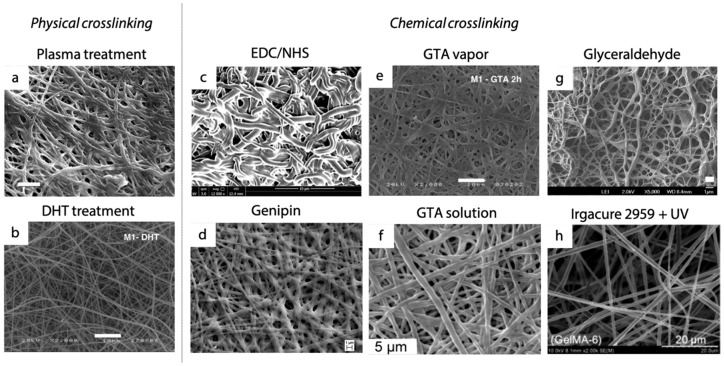
Morphology of electrospun gelatin nanofibers cross-linked with different strategies: (**a**) Plasma treatment (scale bar: 5 μm; reprinted from [71]), (**b**) dehydrothermal treatment (scale bar: 10 μm; reprinted from [54] with permission of Elsevier), (**c**) EDC/NHS (scale bar = 10 μm; reprinted from [72] with permission of John Wiley and Sons), (**d**) genipin (scale bar: 1 μm; reprinted from [78] with permission of Elsevier), (**e**) glutaraldehyde vapor (scale bar: 10 μm; reprinted from [54] with permission of Elsevier), (**f**) glutaraldehyde solution (scale bar: 5 μm; reprinted from [90] with permission of John Wiley and Sons), (**g**) glyceraldehyde (scale bar: 1 μm reprinted from [70] with permission of ACS Publications), and (**h**) Irgacure 2959 with UV treatment (scale bar: 5 μm reprinted from [98] with permission of Elsevier).

Only a few studies reported the use of plasma treatment in the stabilization of electrospun gelatin nanofibers aiming at conferring stability in an aqueous environment (i.e., in vivo physiological fluids) and adequate mechanical properties for their use in tissue engineering applications. For the application of plasma treatment, as-electrospun gelatin matrices are inserted in a plasma chamber in which parameters such as pressure and gas atmosphere can be tuned. The majority of these studies put into evidence that low pressure plasma treatments, operated in oxygen or argon, were not successful in cross-linking gelatin nanofibers. In particular, SEM analyses and weight loss assessments presented by Ratanavaporn et al. [56] and Sisson et al. [70], respectively, highlighted the achievement of a low cross-linking degree that macroscopically resulted in consistent fiber melting, pointing out the failure of this type of physical cross-linking strategy when low pressure is applied during the plasma treatment. More recently, Liguori et al. [71] pointed out an interesting advancement in the field. In fact, they demonstrated that the use of an atmospheric pressure non-equilibrium plasma operated in open air can effectively be considered a suitable approach for the successful cross-linking of gelatin nanofibers, without requiring chemical agents. In their study, plasma treatment triggers an adequate cross-linking reaction, giving structural and morphological stability (Figure 4a) to gelatin ultrafine fibers.

Dehydrothermal treatment is also considered for the stabilization of electrospun matrices. Literature studies propose that gelatin nanofibers are put at relatively high temperatures (140–160 °C), usually under vacuum, for a time that varies from 24 to 72 h [54,56,72]. This strategy allows for preserving a good fiber morphology (Figure 4b), giving stability to the nanofibrous structure. However the degree of cross-linking achieved by DHT is relatively low and a consistent fibers swelling was observed after immersion of matrices in aqueous environment at 37 °C, with consequent loss of the morphology of the electrospun fibers [72]. 

Electron-beam irradiation on electrospun gelatin matrices has been also investigated to promote the electrospun gelatin cross-linking. As-spun gelatin matrices are placed under an electron beam accelerator and parameters such as irradiation dose, accelerating voltage, current, and dose rate are tuned in order to induce the formation of free radicals that lead to the formation of bonds among gelatin chains. Lee et al. [53] tested this strategy, obtaining good results in terms of morphology preservation. However, they highlighted that an excess of irradiation dose may cause a consistent weight loss over time due to the irradiated polymeric chains scission, instead of cross-linking. 

Therefore, as the majority of scientific researches have pointed out, gelatin physical cross-linking strategies results are generally affected by relatively low cross-linking efficiency, leading to relatively lower properties and limited electrospun matrix stability.

### 4.2. Chemical Strategies to Cross-Link Electrospun Matrices

Chemical cross-linking strategies are the most widely used for the stabilization of electrospun gelatin matrices (Table 2). In fact, the use of a chemical cross-linker, both a zero-length or non-zero length type, appears as an efficient strategy for inducing the formation of stable covalent bonds among gelatin polymer chains. This chemical stabilization consequently results in a relatively good preservation of nanofiber morphology and the potentially achievable cross-linking degrees are usually more satisfactory than the ones obtained with the application of physical cross-linking strategies [56]. 

Among the chemical methods described in the previous sections, glutaraldehyde (GTA) vapor is the most widely described for the stabilization of gelatin nanofibrous matrices [54,70,79,80,81,82,83,84,85,86,87,88,89]. The nanofibrous gelatin samples are placed into an air-tight container filled with saturated GTA vapor, where GTA cross-linker molecules lead to the formation of covalent bonds among gelatin polymer chains. In this way, the gelatin nanofibers are cross-linked and the concentration of GTA results a crucial parameter in preserving nanofiber morphology (Figure 4e). Although GTA provides good improvement in mechanical properties and scaffold stability [79], contradictory evidence was highlighted on the cytotoxicity of GTA cross-linked materials [102,103,104]. Strategies proposed to reduce the risk of cytotoxic effects include lowering the concentration of the cross-linker or introducing post treatments (e.g., rinsing, washing with molecules to bind unreacted cross-linker, evaporation in vacuum desiccator) to eliminate potentially toxic byproducts. An additional limitation of this strategy is the difficulty in controlling GTA in the form of vapor, which results in less reproducible cross-linking degrees from test to test. To overcome these drawbacks, several alternative strategies (e.g., immersion) have been tested, with results comparable to those achieved by GTA vapor cross-linking. For example, some researchers [90,91] proposed the use of GTA in a tert-butanol (t-BuOH) solution, to avoid the complexity of GTA vapor control, and induced the cross-linking of gelatin matrices by a simple immersion process. A GTA solution in t-BuOH was used instead of a water-based solution in order to temporally avoid the dissolution of gelatin nanofibers during the cross-linking reaction, activated by an immersion process. They successfully obtained electrospun nanofibrous matrices that, after the GTA cross-linking, remained stable in an aqueous environment up to 15 days. However, they noticed that relatively high concentrations of cross-linker (i.e., 5% v/v tert-butanol solution) determined a partial melting of fibers and an increase of their diameter (Figure 4f). This phenomenon determined a proportional increase in the ultimate tensile stress of nanofibrous matrices with increasing cross-linker concentrations and influenced the response of seeded cells (chondrocytes). After 8 days of culture, enhanced chondrogenesis was observed on the stiffer matrix, where the cell density was found to be higher, reaching the number found in native cartilage. Thus, they demonstrated how the tuning of the cross-linking parameters can affect the mechanical and biological properties of matrices in in vitro tests [90]. Despite the positive outcomes provided by the use of GTA, both in form of vapor and solution, several studies currently aim at providing new cross-linking protocols for electrospun matrices to avoid any possible cytotoxic effects caused by GTA cross-linker residues. 

Analogously exploiting an immersion process, different protocols were proposed using the EDC/NHS strategy. As a zero-length cross-linking agent, EDC allows for the formation of stable covalent bonds without becoming part of the cross-linked gelatin network, thus avoiding potential cytotoxic effects. Different studies [56,58,72,73,74,75] demonstrated its suitability in stabilizing electrospun gelatin nanofibers, even if some challenges regarding the preservation of a desired nanofibrous morphology are still open. A fundamental parameter in the EDC-based cross-linking reaction is the solvent involved. As Chou et al. demonstrated [58], the choice of the solvent determines differences in the interactions between the cross-linker molecules and the polymer chains, thereby affecting the formation of a stable cross-linked network. A mixture of ethanol and water is the most successfully used solvent in carbodiimide cross-linking. Using this mixture, an increase in electrospun fiber swelling was observed in the presence of an excess of water (i.e., 20 vol%), leading to an enlargement of fibers and a change in their morphology and arrangement (Figure 4c). For this reason, better results were obtained by using pure ethanol, a non-aqueous solvent that may reduce the hydrolysis of carbodiimide-activated derivatives, thus improving the cross-linking yields [105]. However, a high concentration (i.e., 99 vol%) of ethanol can limit the solubilization of carbodiimide, negatively affecting the extent of cross-linking [58]. Thus, a balance between these parameters must be considered in order to obtain stable substrates (i.e., medium/high level of cross-linking degree) with suitable nanofibrous morphology. 

A similar immersion process is involved when genipin is selected as the cross-linker. The protocols proposed consider the immersion of as-spun gelatin matrices in a genipin solution, with the possibility to control the cross-linker concentration and the reaction time. Despite its proven biocompatibility, its high cost makes this cross-linker less investigated compared to EDC/NHS or glutaraldehyde for the stabilization of nanofibrous matrices [76,78]. As for carbodiimide chemistry, the majority of the proposed protocols consider ethanol or ethanol/water mixtures for cross-linker solubilization, but involve longer reaction times (i.e., 1–7 days) than common EDC/NHS cross-linking protocols (i.e., 2 h–2 days). The presence of water in the cross-linking bath usually determines a consistent swelling of gelatin nanofibers (Figure 4d) that are not yet cross-linked due to the slow cross-linking kinetic of genipin [70]. Panzavolta et al. [76] widely investigated the effects of genipin cross-linking on the preservation of fiber morphology after the immersion process and, in particular, they evaluated the cross-linking degree, stability, and mechanical properties of electrospun gelatin matrices reached by this cross-linking strategy. They proved that the addition of a small amount of genipin (i.e., genipin concentration <0.6% w/v) to the electrospinning solution remarkably improved the final results, compared with the immersion of as-spun matrices in genipin solution, thus enhancing the maintenance of a good nanofiber morphology. They finally demonstrated that the use of genipin couples a good cross-linking efficiency with a very low toxicity that has been proved by preliminary in vitro studies [76].

Alternatively, it is possible to obtain photo-crosslinkable gelatin (GelMA) fibrous matrices by incorporating reactive methacryloyl groups onto gelatin. If compared with other chemical cross-linking strategies, this strategy requires an additional step for the achievement of a stable gelatin fibrous network. In fact, a chemical modification must be applied to the gelatin polymer chains prior to the electrospinning process in order to introduce the reactive groups required for the UV-driven cross-linking. Zhao et al. [42] demonstrated that, by the adjustment of the UV light exposure time of the as-spun matrices, that, in turn, influences the cross-linking density, the physical properties of the fabricated electrospun matrices (i.e., water retention capacity and stiffness) can be tailored [100]. 

### 4.3. In Situ Cross-Linking Strategies

Physical and chemical cross-linking strategies are typically performed at completion of the fiber fabrication procedure (post-processing cross-linking). However, the majority of chemical cross-linking strategies require the immersion of electrospun matrices in a solvent (water, ethanol, …) that is likely to provoke undesirable morphological changes in the fibrous electrospun structures. In order to overcome this major drawback, some researchers investigated a valid alternative to the post-processing cross-linking strategies by performing cross-linking and fiber spinning simultaneously (generally referred to as in situ cross-linking). UV irradiation, for example, was tested as a possible in situ cross-linking strategy by adding a photoreactive poly (acrylic acid) (PAA) conjugated with azides in the gelatin solution and by applying a UV light to the polymer jet while fibers were forming. As the polymer solution is stretched by the electric field, UV radiation induces the activation of phenyl azido groups into short-lived nitrenes that cause the formation of stable covalent bonds among gelatin polymer chains [101].

Similarly, Nguyen et al. [87] investigated an in situ cross-linking approach using GTA as cross-linker. In order to reduce the possible cytotoxic effects of GTA, they tested this cross-linking agent by adding it in a low amount (i.e., 0.006% w/v) to the gelatin solution immediately before the electrospinning process in order to induce a cross-linking of gelatin fibers during their formation. The use of a strong acid (trifluoroacetic acid) as a solvent for gelatin and GTA solubilization limits the cross-linking effect during the electrospinning process, but allows a sufficient stabilization of the fibrous matrix due to its evaporation at the end of the process. A similar approach, that used 1,4-butanediol diglycidyl ether (BDDGE) for the in situ cross-linking of gelatin fibers, has been considered by Dias et al. [93]. BDDGE is added to the gelatin solution before spinning and it induces the formation of covalent bonds between gelatin chains during the electrospinning process, eventually becoming part of the cross-linked network. The use of this cross-linker allowed for the successful production of stable matrices with a well-defined fiber morphology. In addition, preliminary in vitro biological tests have demonstrated the potential of this strategy as alternative cross-linker for electrospun gelatin. Successful in situ gelatin cross-linking was also obtained by adding γ-glycidoxypropyltrimethoxysilane (GPTMS) to the solution to be electrospun. The oxirane rings on the GPTMS molecules react with the amino groups of the gelatin chains and pendant silanol groups (Si–OH) are formed by the hydrolysis of the trimethoxy groups on the GPTMS through an acid-catalyzed reaction. During solvent evaporation, Si–O–Si bonds are formed through the condensation of two Si–OH. These linkages provide inter-chain covalent bonds, resulting in a cross-linked network. Cross-linking, that it is mainly a condensation reaction, occurs after the fibers are collected, during the solvent evaporation, and does not affect the electrospinning process, leading to the production of homogeneous nanofiber matrices [96,97].

## 5. Biomedical Applications

The advantages and unique features of electrospun gelatin nanofibers have led to a variety of biomedical applications. In fact, the high surface to volume ratio, peculiar porosity, and physicomechanical properties, which can be tuned by varying the process parameters, together with the ECM-mimicking nanofibrous structure, make electrospun gelatin fibers particularly suitable for tissue engineering and drug delivery applications [106]. These applications include skin regeneration and wound healing [54,85,86,93,98,100], vascular tissue engineering [82,83], and nerve regeneration [96,97].

**Figure 5:**
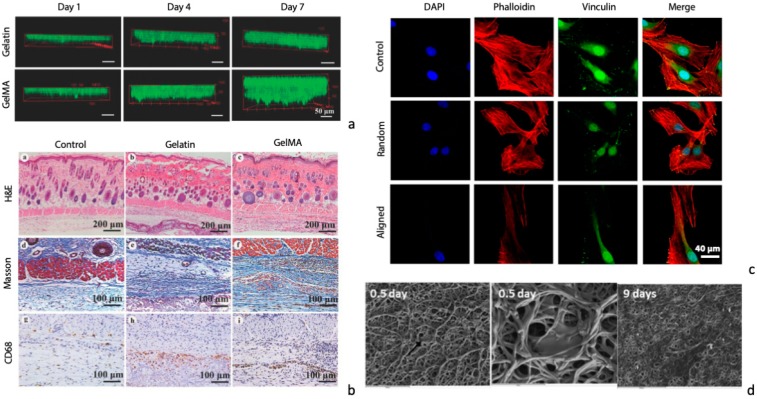
Tissue engineering applications of electrospun cross-linked gelatin matrices. (**a**) Cell infiltration inside electrospun gelatin vs. GelMA scaffolds (phalloidin staining, Alexa Fluor 488; scale bar: 50 μm) and (**b**) in vivo assessment of electrospun gelatin and GelMA scaffold skin regenerative potential by histological analysis (scale bar: 200 and 100 μm); reprinted from [100] with permission of Elsevier. (**c**) Confocal images of primary Schwann cells cultured on random and aligned electrospun gelatin matrices (vs. tissue culture plastic as control; scale bar: 40 μm) [97]. (**d**) SEM micrographs of human umbilical vein smooth muscle cells cultured on electrospun gelatin matrices for smooth muscle regeneration in vascular tissue engineering; reprinted from [83] with permission of John Wiley and Sons.

Regeneration of skin requires a matrix that allows for cell infiltration and proliferation to accelerate wound healing and to limit possible complications due to an inefficient tissue regeneration. Different authors have shown that the use of flat electrospun gelatin matrices can successfully promote the regeneration of skin after traumatic/pathological events. For instance, electrospun fish gelatin cross-linked by GTA vapor, genipin, or dehydrothermal treatment showed 3T3 murine fibroblast cells adhesion and proliferation, until confluence on the colonized electrospun matrix was reached [54]. A glutaraldehyde-cross-linked electrospun gelatin matrix was proved to be an optimal substrate for in vitro colonization by human dermal fibroblasts, a more representative cell model, that successfully fully colonized the matrix, although their penetration was limited to the surface of the scaffold [85]. Other authors [98,100] showed the possibility of an in depth colonization of the electrospun matrix, likely related to the different mechanical properties achieved by controlling cross-linking strategies and process parameters. In fact, softer fibers successfully promoted a deeper cell colonization in the scaffold, compared to stiffer materials (Figure 5a). Similarly, human dermal neonatal fibroblasts were shown to fully colonize a BDDGE-cross-linked electrospun gelatin matrix and to deposit fibronectin after 7 days of culture, which is fundamental for a functional skin regeneration [93]. Given the promising in vitro results, the potential application of the electrospun gelatin matrices was also investigated in in vivo animal models. Glutaraldehyde-cross-linked electrospun gelatin matrices proved their potential for wound regeneration in a rat model, although a lower regenerative effect was observed compared to chitosan matrices [85]. Electrospun photo-crosslinkable gelatin matrices (GelMA) proved their potential in in vivo skin regeneration. Authors were able to promote cells adhesion and tissue infiltration by modulating the physico-mechanical properties of the electrospun matrices to obtain soft and elastic fibrous scaffolds for wound healing [100]. After 3 weeks from the scaffold application onto the wound, electrospun GelMA scaffolds completely healed the wound, compared to glutaraldehyde cross-linked gelatin matrices and electrospun PLGA scaffolds, where >5% of the wound area was still observed after 3 weeks [100]. Moreover, GelMA membranes were successfully replaced by dermal tissue containing blood vessels (Figure 5b), a fundamental achievement for the long-term survival of the regenerated tissue. Finally, given the high risk of infections related to wounds, the possibility of adding antibacterial functionality to the scaffolds by loading AgNO_3_ in the electrospun gelatin matrices, to prevent bacterial infection during the wound healing process, was also demonstrated [86].

For nerve regeneration, tubular structures can be fabricated by electrically collecting drive fibers on rotating cylindrical mandrels [92]. Engineered neural stem-like cells successfully adhered, proliferated, and penetrated in glyceraldehyde-cross-linked gelatin matrices co-electrospun with PLLA, proving the potential use of such a scaffold to guide peripheral nerve regeneration. Moreover, controlling the orientation of electrospun fibers allowed for guiding the biological response of Schwann cells that successfully orientated along aligned electrospun gelatin fibers (Figure 5c) [97].

The use of rotating targets for the collection of the fibers during the electrospinning process is also an attractive possibility for the production of grafts for vascular tissue engineering. Tubular electrospun matrices can, in fact, be fabricated with different features, including the diameter of the cylindrical structure, porosity, stiffness and fiber orientation, parameters that, in turn, heavily influence the biological performance of the scaffolds. Glutaraldehyde vapor-cross-linked electrospun gelatin scaffolds were shown to promote in vitro human umbilical vein smooth muscle cells (Figure 5d) [83] and human smooth muscle cell [82] adhesion and proliferation. Cells were able to elongate following the preferential orientation of electrospun fibers, thus proving their ability in mediating cells response. Moreover, higher proliferation rates were obtained by dynamic culturing of cells in a rotary bioreactor [83] to further support the use of electrospun gelatin tubular scaffolds as tissue engineering vascular grafts.

## 6. Concluding Remarks and Future Perspectives

The combination of gelatin with electrospinning fabrication results in a unique solution with outstanding features for tissue engineering purposes. In fact, gelatin offers cell-adhesive motives, together with tunable physicochemical properties and reduced costs [11]. At the same time, its electrospinning allows for easy fabrication of morphologically ECM-biomimetic scaffolds. The use of electrospun gelatin thus allows for the fabrication of biomimetic/cell-instructive scaffolds, both in terms of chemical interaction between cells and the biomaterial (i.e., gelatin cells adhesive motifs, degradability and scaffold/ECM remodeling) and in terms of nano-topographical features that can guide cell response and control cell adhesion and migration in an ECM-biomimetic microenvironment [4].

**Figure 6:**
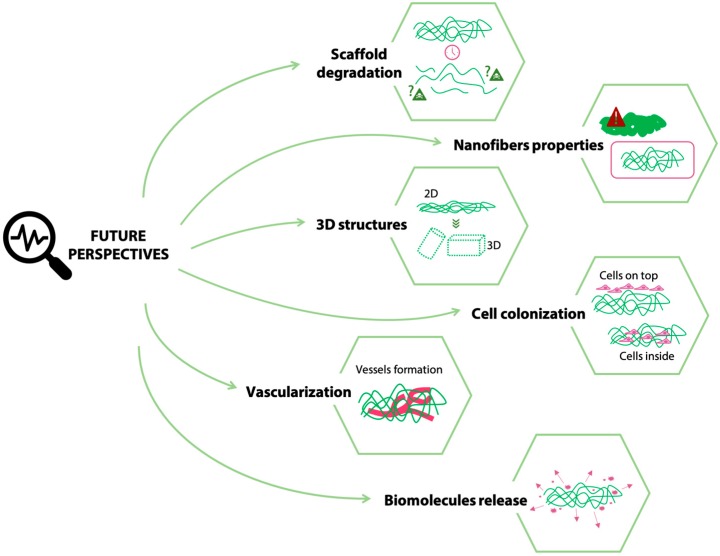
Future perspectives and open challenges in the fabrication of electrospun gelatin scaffolds.

While the optimization of electrospinning parameters to obtain an optimized electrospun matrix (i.e., reproducible beads-free electrospun matrix) does not generally encounter significant obstacles, the development of an efficient cross-linking strategy to stabilize the obtained structure is more challenging. This latter fabrication step is, in fact, crucial and underestimated. The insufficient maintenance of electrospun fiber morphology during cross-linking can impair the benefit of processing by electrospinning and reduce the effectiveness of matrices for tissue engineered purposes. Different well-established strategies have been proposed to cross-link gelatin as a scaffolding material, as described in Section 3. Most of these methods were also successfully applied to cross-link electrospun gelatin matrices. Both physical (i.e., high-energy electron beam, plasma treatment, and DHT) and chemical (i.e., EDC/NHS, genipin, glutaraldehyde, glyceraldehyde, BDDGE, isocyanates, procyanidin, oxidized sucrose, GPTMS, and GelMA) cross-linking strategies were extensively applied to gelatin in the form of fibers, with satisfactory results. The only exception, to date, is probably represented by enzymatic cross-linking methods, to which only little attention was dedicated. Despite the advantages associated with this strategy [50,51,52], only a few works described their use for electrospun gelatin matrices [107] and further investigations are required. Moreover, most works [61,72,100] prove, either by in vitro or in vivo tests, the cytocompatibility and biocompatibility of the electrospun gelatin matrices crosslinked by different methods. However, they describe relatively short-term results (i.e., days or weeks). Thus, the possible cytotoxic effects of degrading gelatin scaffolds, at longer times, are generally not considered, since the experimental times do not allow for complete scaffold degradation. Proving the absence of cytotoxic effects during (and at the completion of) electrospun gelatin scaffold degradation is fundamental since, after implantation and degradation, the safety of degradation products must be assured in the long term to allow for an adequate in vivo disposal of the scaffolds’ degradation products (Figure 6).

When cross-linking electrospun gelatin, compared to gelatin hydrogels, the additional challenge is to provide adequate conditions to maintain the fibrous architecture. Extensive swelling of fibers in the cross-linking solution and inter fiber cross-linking, in fact, can lead to a loss of nano-morphology and the obtainment of undesired film-like surfaces. Accordingly, the cross-linking time, the solvent, and the cross-linker concentration must be finely tuned. Most of the papers focusing on the cross-linking of electrospun gelatin show the nanofibers morphology only immediately after the electrospinning/cross-linking procedure [56,58,70,78,81,86,100], which is preserved to different extents according to the chosen method. On the other hand, very few studies consider the maintenance of fiber morphology in physiological-like conditions (i.e., aqueous environment at 37 °C), which is actually another fundamental issue for the final application of electrospun scaffolds [72,99]. Large swelling in water can still compromise the cross-linked fiber structure together with the porosity of the matrices and can affect, in turn, the biological response. The extent of cross-linking and the initial morphology of the scaffold are the most important parameters and, by their accurate control, the morphologically favorable structure of electrospun fibers can be preserved to guarantee a suitable nano-topography to allow cells migration and enhance their proliferation and ECM remodeling, thus achieving the desired tissue regeneration. Investigations and proof of the preservation of the electrospun gelatin matrices after immersion in an in vivo-like environment and its effect on cell behavior thus represents a point that still needs to be addressed (Figure 6). Most of the authors [72,76,94] vary the extent of crosslinking of the electrospun gelatin fibers to tune their morphology and stability; however, it should be considered that a variation in the extent of crosslinking also results in a modification of the mechanical properties, which in turn can influence the cell response. Thus, an appropriate balance between the extent of crosslinking, mechanical properties, morphology, and cell response should be finely addressed.

Despite the great achievements in the tissue engineering field, the use of electrospun gelatin matrices as scaffolds is currently mostly investigated for relatively limited applications (i.e., mostly skin [93,98], nerve regeneration [96,97], and vascular tissue engineering [82,83]). Future challenges that will allow for expansion of the applications of electrospun gelatin matrices include the fabrication of 3D structures and strategies that allow for the distribution of cells throughout the thickness of the electrospun scaffold. In fact, electrospun gelatin matrices are generally obtained as 2D matrices and the thickness of the obtained scaffolds is generally limited at the order of micrometers [108]. The development of complex 3D collectors, recently investigated for the electrospinning of other polymers [109,110], still needs to be proved for gelatin and would expand the array of its possible biomedical applications (Figure 6). As the thickness of the obtained scaffolds increases, methods to promote a full-thickness cell colonization should be developed. In fact, the colonization of electrospun gelatin matrices by cells is still challenging and it has been demonstrated to depend on the mechanical properties of the electrospun gelatin matrices [100]. The achievement of a full-thickness colonization by cells (Figure 6) is strictly related to the design of an appropriate pore size and distribution that should be suitable for cell infiltration. Possible methods to be investigated to increase the pore size dimension of electrospun gelatin matrices rely on the use of co-electrospinning of sacrificial fibers or particles (e.g., polyethylene oxide) [111,112], to be removed upon the completions of the electrospinning process. In parallel, as the thickness increases and the cells are distributed in the 3D scaffold, studies on the possible vascularization of the obtained structures must be conducted to ensure cell survival after the in vivo implant of the scaffold (Figure 6). Finally, an improved cell response and tissue regeneration might be achieved by the addition of bioactive molecules, such as drugs and growth factors, to the electrospun gelatin scaffolds [78,81]. Studies on the relationship between the extent of crosslinking of electrospun gelatin matrices and the release profile of such bioactive molecules would allow for a fine and improved tissue regeneration.

## Figures and Tables

**Table 1:** Examples of cross-linking methods used for the production of gelatin hydrogels. The cross-linking methods are divided in three categories, physical, chemical, and enzymatic. For each example, the cross-linking methods, gelatin type, and gelatin concentration used are reported.

Cross-Linking Method	Gelatin Type	Gelatin Concentration (w/v)	Reference
**Physical Methods**			
High energy electron beam	type A	2–20%	Wisotzki et al. 2014 [22]
	type B	10%	Van Vlierberghe 2016 [23]
	type A, type B and cold fish skin gelatin	1–30%	Terao et al. 2012 [24]
γ-irradiation	type A	3%	Cataldo et al. 2008 [25]
	type B	1–20%	Kojima et al. 2004 [26]
Plasma treatment	type A	1.25–2.5%	Prasertsung et al. 2013 [27]
Dehydrothermal treatment	type A	3%	Hussain et al. 2014 [28]
	type B	10%	Omata et al. 2014 [29]
	type A	10%	Prasertsung et al. 2010 [30]
**Chemical Methods**			
EDC/NHS ^1^	type A and B	10%	Kuijpers et al. 2012 [31]
	type A	10%	Claaßen et al. 2017 [32]
	type B	3%	Rodriguez et al. 2016 [33]
	type B	10%	Gorgieva et al. 2014 [34]
Formaldehyde	type B	2%	Ninan et al. 2013 [35]
Glutaraldehyde	-	5%	Fan et al. 2018 [36]
	type B	20%	Poursamar et al. 2016 [37]
Genipin	type A	2–10%	Kirchmajer et al. 2013 [38]
	-	10%	Wu et al. 2013 [39]
	-	8%	Liang et al. 2004 [40]
	-	10%	Focaroli et al. 2014 [41]
Irgacure 2959 + UV light ^2^	type B	5–20%	Zhao et al. 2016 [42]
	type B	10%	Van Nieuwenhove et al. 2016 [43]
	type B	10%	Zhou et al. 2014 [44]
	type A	5–10%	Celikkin et al. 2017 [45]
*N*,*N*′-methylenebis (acrylamide)	type A	15–25%	Contessi Negrini et al. 2019 [46,47]
Isophorone diisocyanate	-	6%	Subramanian et al. 2013 [48]
Ethylene glycol diglycidyl ether	type B	15%	Vargas et al. 2008 [49]
**Enzymatic Methods**			
Microbial transglutaminase	type A	10%	Yung et al. 2007 [50]
	type A	4%	Broderick et al. 2004 [51]
	type A	1–10%	Yang et al. 2016 [52]

^1^ 1-ethyl-3-(3-dimethylaminopropyl) carbodiimide/N-hydroxysuccinimide. ^2^ Gelatin was chemically modified by methacrylate groups, subsequently cross-linked by using a photo initiator and UV light.

**Table 2:** Cross-linking strategies employed in the stabilization of electrospun gelatin nanofibers. Strategies are divided by physical and chemical methods. The type of gelatin and the main parameters involved in the cross-linking process are reported, as well as the final aim of the study.

**Physical Methods**							
	**Gelatin**	**Parameters**				**Application**	**Reference**
		**Irradiation Dose**	**Accelerating Voltage**	**Current**	**Dose Rate**		
**Electron Beam Irradiation**	type B	10–300 kGy	1 MeV	17 mA	8.33 kGy/s	Soft tissue engineering	Lee et al. [53]
	**Gelatin**	**Parameters**				**Application**	**Reference**
		**Pressure**	**Gas**	**Reaction Time**	**Type of Plasma**		
**Plasma Treatment**	-	Low	Oxygen	2 min	Non-equilibrium	Tissue engineering	Sisson et al. 2009 [70]
	type A, B	Low	Argon	-	Non-equilibrium Pulsed inductively coupled	Biomedical	Ratanavaraporn et al. 2010 [56]
	type A	Atmospheric	Air	20 min	Non-equilibrium	Tissue engineering	Liguori et al. 2016 [71]
	**Gelatin**	**Parameters**				**Application**	**Reference**
		**Reaction Temperature**		**Reaction Time**			
**Dehydrothermal Treatment**	type A, B	140 °C		48 h		Biomedical	Ratanavaraporn et al. 2010 [56]
	Fish	140 °C		24/48/72 h		Tissue engineering	Gomes et al. 2013 [54]
	type A	160 °C		48 h		Tissue engineering	Ghassemi and Slaughter 2018 [72]
**Chemical Methods**							
	**Gelatin**	**Parameters**				**Application**	**Reference**
		**Cross-Linker Concentration **	**Solvent**	**Reaction Time**	**Reaction Temperature**		
**EDC/NHS**	type A	EDC = 50 mM	EtOH/dH_2_O 8/2	24 h	4 °C	Biomedical	Li et al. 2006 [73]
	-	EDC = 5/25/50/75 mM EDC/NHS = 2.5/1	EtOH/dH_2_O 9/1	24 h	4 °C	Periodontal tissue regeneration/Cornea regeneration	Zhang et al. 2009 [74] Tonsomboon et al. 2013 [75]
	type A, B	EDC = 14 mM NHS = 5.5 mM	EtOH or dH_2_O	2 h	-	Biomedical	Ratanavaraporn et al. 2010 [56]
	type B	EDC = 2 M NHS = 1 M	EtOH/dH_2_O 9/1	7 h	-	Tissue engineering	Ghassemi and Slaughter 2018 [72]
	type A	EDC = 5 mM/mg sample EDC/NHS = 5/1	EtOH/dH_2_O (80–99.5 vol%)	48 h	25 °C	Ophthalmic	Chou et al. [58]
**Genipin**	type A	5–7%	EtOH	3–7 days	37 °C	Tissue engineering	Panzavolta et al. 2011 [76]; Chen et al. [77]
	-	0.1–0.5%	EtOH	3 days	37 °C	Angiogenesis in Tissue engineering	Del Gaudio et al. 2013 [78]
	-	0.1–2%	EtOH/dH_2_O 70%	19 h	-	Tissue engineering	Sisson et al. 2009 [70]
	Fish	2%	EtOH/dH_2_O 90%	1–5 days	-	Tissue engineering	Gomes et al. 2013 [54]
**Glutaraldehyde Vapor**	type A	25%	10 mL dH_2_O	6–12 h 1–2–3–4 days	-	Biomedical	Zhang et al. 2006 [79]; Vardiani et al. 2019 [80]; Chen et al. 2012 [77]
	-	0.5%	-	19 h	-	Tissue engineering	Sisson et al. 2009 [70]
	type A	25%	20 mL dH_2_O	2–4–6–8–10 min	-	Drug delivery	Laha et al. 2016 [81]
	type A	10%	-	2 h	-	Vascular tissue engineering	Y. Elsayed et al. 2016 [82]
	type A	25%	-	2 h	-	Vascular tissue engineering	Yahya Elsayed et al. 2016 [83]
	Fish	2.5%	In situ	8 h	-	Tissue engineering	Zhan et al. 2016 [84]
	Fish	5%	10 mL dH_2_O	1–24 h	40 °C	Tissue engineering/Skin regeneration	Gomes et al. 2013 [54,85]
	type A	50%	20 mL dH_2_O	1–3 h	37 °C	Wound healing	Rujitanaroj et al. 2008 [86]
	type A	0.05%	In situ	-	-	Tissue engineering	Nguyen et al. 2010 [87]
	-	50%	-	3–24 h	-	Drug delivery	Lakshminarayanan et al. 2014 [88]
	-	50%	-	15–45–90–360 min	25 °C	Tissue engineering	Wu et al. 2011 [89]
**Glutaraldehyde Solution**	type B	0.1–5%	10 mL t-BuOH	1 h crosslink + freeze-dry	30 °C	Cartilage tissue regeneration	Skotak et al. 2010 [90]; Skotak et al. 2011 [91]
**Glyceraldehyde**	-	0.1–0.5%	EtOH 70%	19 h	Room temp	Tissue engineering	Sisson et al. 2009 [70]
	Gelatin + PLLA	0.5–0.7%	EtOH 70%	19 h	Room temp	Nervous tissue regeneration	Binan et al. 2014 [92]
**1,4-Butanediol Diglycidyl Ether (BDDGE)**	type A	2–4–6%	In situ	24–48–72 h	Room temp	Skin regeneration	Dias et al. 2017 [93]
**Hexamethylene Diisocyanate**	type A	1x, 5x, 10x ratio of isocyanate/amine	In situ	3 h	Room temp	Tissue engineering	Kishan et al. 2015 [94]
**Procyanidine**	type A	0.5–1–2–3–4–5%	EtOH 75%	1–6 days	20–30–40–50–60 °C	Tissue engineering	Chen et al. [77]
**Oxidized Sucrose**	type A	0.1–0.5–1–2%	EtOH	1–3–5 days	37 °C	-	Jalaja et al. 2015 [95]
	**Gelatin**	**Parameters**				**Application**	**Reference**
		**Cross-Linker Concentration**		**Reaction Time**			
**γ-Glycidoxypropyltrimethoxysilane (GPTMS)**	type A	92 μL/gram_gelatin_		In situ		Peripheral nerve regeneration	Tonda-Turo et al. 2013 [96]; Gnavi et al. 2015 [97]
**Irgacure 2959 + UV Light**	type A	10%		30 min (immersion + UV light)		Skin regeneration	Sun et al. 2017 [98]
	GelMA + PCL	0.015%		20 min (immersion) 10 min/side (UV light)		Tissue engineering	Ferreira et al. 2017 [99]
	type A	10%		2 h (immersion) 2–6–10 min/side (UV light)		Wound healing	Zhao et al. 2017 [100]
	**Gelatin**	**Parameters**				**Application**	**Reference**
**UV**	Gel + Poly (acrylic acid-g-azidoaniline)	Two UV lamp (18 W) during electrospinning process (in situ)				Tissue engineering	Lin and Tsai 2013 [101]
